# Conspiracy beliefs, COVID-19 vaccine uptake and adherence to public health interventions during the pandemic in Europe

**DOI:** 10.1093/eurpub/ckad089

**Published:** 2023-06-08

**Authors:** Luca Regazzi, Alberto Lontano, Chiara Cadeddu, Pasquale Di Padova, Aldo Rosano

**Affiliations:** Department of Life Sciences and Public Health, Università Cattolica del Sacro Cuore, Largo Francesco Vito 1, Rome 00168, Italy; Department of Life Sciences and Public Health, Università Cattolica del Sacro Cuore, Largo Francesco Vito 1, Rome 00168, Italy; Department of Life Sciences and Public Health, Università Cattolica del Sacro Cuore, Largo Francesco Vito 1, Rome 00168, Italy; National Institute for the Analysis of Public Policies (INAPP), Rome, Italy; National Institute for the Analysis of Public Policies (INAPP), Rome, Italy

## Abstract

**Background:**

Conspiracy beliefs can be a major hindrance causing a lack of compliance with public health measures, including vaccination. We examined the relationship between individual attitudes, socio-demographic factors, conspiracy beliefs, COVID-19 vaccine hesitancy and preferences about pandemic policies in Europe.

**Methods:**

We used data from the 10th round of the European Social Survey, conducted in 2021–22 in 17 European countries. Both a conspiracy index and a personal attitude index for each participant were built by using a Latent Class Analysis model. Then, we used a multilevel regression model to investigate the relationship between a personal attitudes index, socio-demographic factors and country of residence, and a conspiracy index. We descriptively analyse the relationship between the conspiracy index and four main items related to COVID-19.

**Results:**

We found that a higher probability of believing in conspiracy theories was associated with male gender, middle age, lower levels of education, unemployment, lower levels of trust and satisfaction and right-wing political orientation. The country of residence was a contextual factor, with eastern European countries having higher levels of conspiracy beliefs. Individuals who expressed conspiracy beliefs had lower COVID-19 vaccine uptake, were less satisfied with the way health services coped with the pandemic and less supportive of governmental restrictions.

**Conclusions:**

This study provides valuable insights into the factors associated with conspiracy beliefs and their potential impact on public health. The findings highlight the need for effective strategies to address the underlying factors driving conspiracy beliefs, reduce vaccine hesitancy and promote acceptance of public health interventions.

## Introduction

The association between vaccine hesitancy and conspiracy beliefs has been shown in previous literature.^1^ However, it is still not clear if the latter has an additional effect on refusing COVID-19 vaccination.

Conspiracy theories represent the ‘proposed explanation of events that point to a small group of people (the conspirators) acting in secret for their own benefit, against the common good, as the main causal factor’.^2^ They are adopted where important psychological needs are being met, for instance in conditions of anxiety, insecurity or threat (e.g. political instability or outbreak of pandemics).^3^^,^^4^ Individuals who feel more politically powerless are more susceptible to conspiracy theories and are also more likely to accept information from sources that question the legitimacy of the political system.^5–7^ In this sense, the COVID-19 pandemic has been the perfect breeding ground for conspiracy theories.^8^

Literature on this topic indicates that conspiracy attitudes are associated with individual socio-demographic characteristics (such as gender, age, education, residence, occupational status, professional position, etc.), and a trend consistent with the attitudes, trust and credibility placed in institutions, fellow human beings, the world of science and politics has been shown.^8^^,^^9^

A link has been highlighted between conspiracy beliefs and extremism as well as anti-democratic attitudes since they act as a ‘radicalizing multiplier’ and can lead to estrangement from the rest of the community.^10^

Evidence that highlights that conspiracy theories pose a major threat to public health, being associated with an increased tendency to resist the recommendations of authorities and public health institutions, neglect of infection prevention behaviours and decreased tendency to vaccinate, is growing.^11^^,^^12^ This was particularly evident during the COVID-19 pandemic, during which they contributed to a climate of distrust toward containment measures in a mentally worn out population.^7^

In this regard, Chen *et al*.^4^^,^^13^ reported how Chinese mothers’ exposure to conspiracy theories was associated with reduced intention to vaccinate their children and to receive HPV vaccination, while Jolley *et al*.^4^^,^^14^ highlighted how marginalized communities of British homosexual males showed greater tendency to develop a conspiracy attitude and reduced their acceptance of pre-exposure prophylaxis.

Consequently, the study of the degree to which conspiracy beliefs are widespread in a population could be of uttermost importance in predicting the level of adherence to vaccination campaigns and, more generally, for health policy implications or provisions adopted by the authorities.^7^^,^^11^

To date, several instruments have been developed to assess belief in conspiracy theories,^15^ such as the Generic Conspiracist Beliefs (GCB) scale,^16^ the Conspiracy Mentality Questionnaire^17^ and the Belief in Conspiracy Theories Inventory.^18^ In 2016, Shapiro *et al*.^19^ proposed a scale measuring the level of conspiracy beliefs related to vaccination—the Vaccine Conspiracy Belief Scale (VCBS). However, it is not specific to COVID-19, which introduced peculiar aspects in terms of beliefs, attitudes and fears.

The European Social Survey (ESS) has been established in 2001 as a research-driven cross-national survey, conducted every 2 years, to gauge the attitudes, beliefs and behavioural patterns of diverse populations in 40 nations.^20^ ESS aims to operate as a research infrastructure that provides superior quality data, measuring the changes (and/or stability) over time within and between European countries in their social structure, living conditions, public opinion and attitudes.^20^

Moreover, a specific section of the ESS 10 addresses COVID-19 conspiracy beliefs and government rule compliance, trying to understand why and how conspiracy theories become more or less prevalent.^21^

The present study aims to investigate the relationship between COVID-19 conspiracy beliefs, personal attitudes and individual socio-demographic characteristics; and between COVID-19 conspiracy beliefs, COVID-19 vaccine uptake and preferences about the policies adopted during the pandemic in 17 European countries.

## Methods

### Data source

The ESS is a biennial international survey that has involved as many as 40 countries since 2001.^20^ It is coordinated by the ESS European Research Infrastructure Consortium (ERIC), and its purposes include monitoring trends in attitudes and values in European countries, consolidating measurement methodologies in cross-national surveys in Europe and developing a set of European social indicators, including indicators of attitude. Between September 2020 and May 2021, the field phase of Round 10 of the ESS took place, involving 32 countries. Among them, 17 countries adopted an optional form containing questions designed to explore beliefs, attitudes and behaviours related to the COVID-19 pandemic. The countries that included such a module in their questionnaires were: Bulgaria (BG), Switzerland (CH), Czechia (CZ), Estonia (EE), Finland (FI), Greece (GR), Croatia (HR), Hungary (HU), Iceland (IS), Italy (IT), Lithuania (LT), North Macedonia (MK), Netherland (NL), Norway (NO), Portugal (PT), Slovenia (SI) and Slovakia (SK).

### Variables selection

The ESS 10 COVID-19 module includes three questions to detect the intensity of conspiracy beliefs, using a 5-point Likert-type response ranging from 1 (maximum agreement) to 5 (maximum disagreement):

1. Groups of scientists manipulate, fabricate or suppress evidence in order to deceive the public.
2. A small secret group of people is responsible for making all major decisions in world politics.
3. Coronavirus is the result of deliberate and concealed efforts of some government or organization.

These items were selected from Brotherton et al. conspiracy battery,^16^ to measure three dimensions of conspiratorial thinking: general conspiracies, more domain specific-scientific coverup and COVID-specific belief. The frequency distributions of the three items by level of agreement (Supplementary table S1a) and by country (Supplementary table S1b) are reported in the Supplementary appendix. The value of Cronbach’s Alpha index (0.81) confirms the internal consistency of the items composing the conspiracy scale.

Personal attitudes were investigated through three main dimensions:

1. trust in science/scientists, people and institutions (11 items);
2. interest in politics and the possibility of exercising a role (5 items);
3. satisfaction in life, economy, government, democracy, education system and health care system (8 items).

Selected socio-demographic characteristics were gender, age, education, occupational status, and economic situation. Education was classified as lower secondary or lower, upper secondary and tertiary; occupational status as employed, unemployed and inactive; the economic situation was classified as comfortable, fair, difficult and very difficult according to a self-evaluation (see Supplementary appendix table S2).

### Statistical analysis

In the first step, latent class analysis (LCA)^22^ was used to define both a conspiracy index and an attitude index. The conspiracy index was built by LCA analysis on the three selected items, which identify the COVID-19 conspiracy beliefs. The index corresponds to the respondents’ probability of belonging to a certain latent class according to the levels of agreement with the conspiracy statements. The appropriate number of classes was chosen through a likelihood-based test. Similarly, an index expressing individual attitudes was constructed based on an LCA conducted on the variables identifying levels of trust, interest in politics and satisfaction in life and public services offered by the country of residence. The classes generated by the LCA allow for grouping respondents according to their levels of trust and satisfaction in the selected dimensions. The dataset contained 3827 records (12.7%) with missing information on at least one of the three items identifying the COVID-19 conspiracy beliefs. Because of the iterative nature of the expectation-maximization (EM) algorithm^23^ adopted to estimate the latent class model, the estimation was feasible even when some of the observations on the variables are missing. In case the answers to the three key questions are simultaneously missing (760 records—2.5%), the EM algorithm imputes, as the probability of belonging to the latent classes, the mean values. Statistical analysis was conducted using SPSS software (version 27.0).

In the second step, we analysed the relationship between conspiracy beliefs (the outcome variable), personal attitudes and individual socio-demographic characteristics (see Supplementary appendix table S2). To take into account the country-level source of variation, a mixed-effects multilevel model^24^ was used to estimate the membership probability to the conspiracy group, considering socio-demographic characteristics and attitudes of respondents. The intraclass correlation coefficient (ICC) was used to test the appropriateness of the multilevel approach. The ICC varies from +1, when group risks differed, but there is no variation within any group, to −1/(*n* − 1) when group means are equal, but the within-group variation is large.^25^

In the third step, we analysed the association between the conspiracy index, COVID-19 vaccine uptake and preferences about the policies adopted during the pandemic in the 17 European participating countries. The vaccine uptake was investigated through the item ‘Whether respondent will get vaccinated against COVID-19 with a vaccine approved by national regulatory authority’. For each country, the percentage of those who responded ‘Yes I have already done’ was compared with the official data on vaccination coverage available through the site ‘Ourworldindata’ and European Centre for Disease Prevention and Control (ECDC) data at the time of the interview in the country, showing a strong agreement (see Supplementary appendix sable S3). Vaccine non-acceptance was measured through the percentage of respondents who answered they would not get vaccinated.

## Results

### Step 1

#### Building a conspiracy index

Through an LCA, three response profiles were identified corresponding to the low (class 1), medium (class 3) and high (class 2) degrees of agreement with the three questions that detect the conspiracy attitude. Class 2, which includes about a quarter of the respondents, is the group with the greatest tendency to approve conspiracy statements. We can therefore use the probability of belonging to class 2 identified with the LCA as a numeric index of conspiracy, whose values range from a minimum of 0 to a maximum of 1.

Supplementary table S4 contains the item response probabilities for the three-class model. Members of the first class were labelled ‘low degree of conspiracy beliefs’ (latent class prevalence 32.4%). Members of class 2 were labelled ‘high degree of conspiracy beliefs’ (latent class prevalence 23.9%). Members of class 3 were those with a moderate degree of conspiracy beliefs (latent class prevalence 43.7%) and constitutes the larger group.

#### Building an attitude index

Through the LCA analysis, we summarized the set of attitudes which, according to the examined literature, seem to be associated with the conspiratorial system of beliefs. We considered the following domains:

1. Trust in science/scientists, people and institutions.
2. Interest in politics and the possibility of exercising an active role.
3. Satisfaction with life, the economy, government, democracy, the education system and the healthcare system.

**Figure 1:**
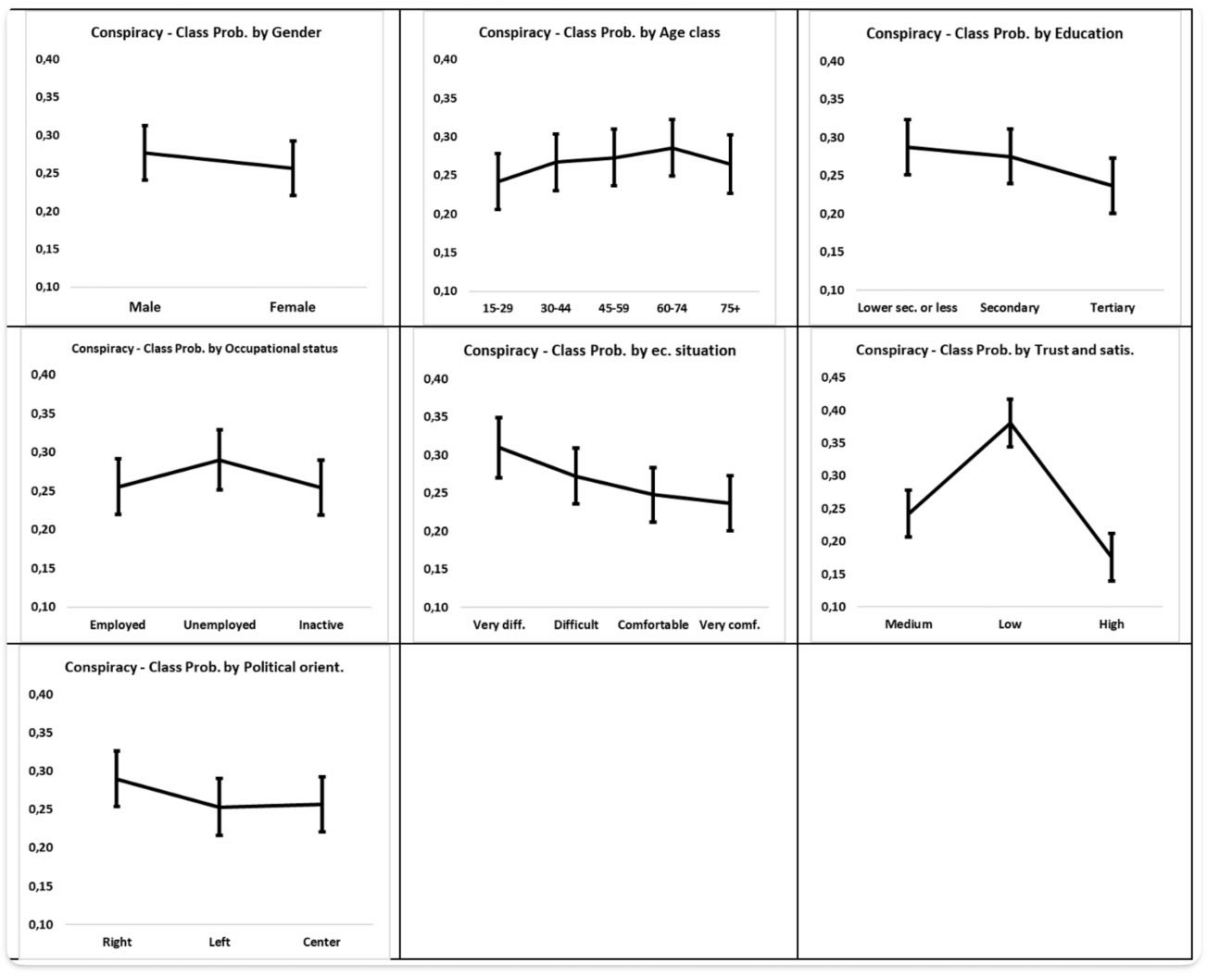
Estimated marginal means for significant fixed effects

Three latent classes with a defined response profile were identified. Figure 1 graphically displays a plot of the class membership profiles through standardized item responses: class 3 collects the interviewees who have on average the highest scores on all items, class 2 the ‘low’ response profiles and class 1 the average profiles (figure 1).

### Step 2

Through a multilevel regression model that takes into account the heterogeneity between countries, we investigate the relationship between the conspiracy index (outcome variable), and the explanatory factors, i.e. the attitude index and socio-demographic variables (sex, age, education, occupational status, perceived economic status and political orientation) (table 1).

**Table 1:** Multilevel regression estimates for the probability of belonging to class of conspiracy believers: standard linear model (model 0), multilevel models: model 1: only country, model 2: model 1 + socio-demographic variable, model 3: model 2 + attitudes, model 4: model 3 + political orientation

Variables		Model 0	Model 1	Model 2	Model 3	Model 4
Sex	Male			0.022***	0.024***	0.020***
	Female (ref)					
Age class	15–29			−0.021**	−0.008	−0.023**
	30–44			0.013	0.017*	0.002
	45–59			0.017*	0.020**	0.008
	60–74			0.025***	0.027***	0.021**
	75+ (ref)					
Education	Lower secondary or lower			0.068***	0.050***	0.051***
	Upper secondary			0.051***	0.041***	0.039***
	Tertiary (ref)					
Occupational status	Employed			0.004	0	0.001
	Unemployed			0.049***	0.034***	0.036***
	Inactive (ref)					
Perceived economic status	Very difficult			0.121***	0.083***	0.073***
	Difficult			0.062***	0.039***	0.036***
	Fair			0.026***	0.015***	0.011*
	Comfortable (ref)					
Trust and satisfaction attitude	Class 1 (medium)				0.059***	0.066***
	Class 2 (low)				0.186***	0.205***
	Class 3 (high) (ref)					
Political orientation	Right					0.034***
	Left					−0.003
	Center (ref)					
						
Intercept		0.201***	0.230***	0.135***	0.083***	0.082***
Variance components	Individual	0.052	0.049	0.047	0.045	0.043
	Country		0.013	0.011	0.006	0.005
−2 log likelihood		37,134,619	35,197,897	33,404,005	32,374,612	26,333,848
AIC		37,136,619	35,201,897	33,408,006	32,378,613	26,337,849
BIC		37,144,931	35,218,521	33,424,560	32,395,167	26,354,110
ICC			0.21	0.19	0.12	0.11
*N*		30,096	30,096	29,086	29,086	25,119

***P < 0.001, **P < 0.01, *P < 0.05.

**Figure 2:**
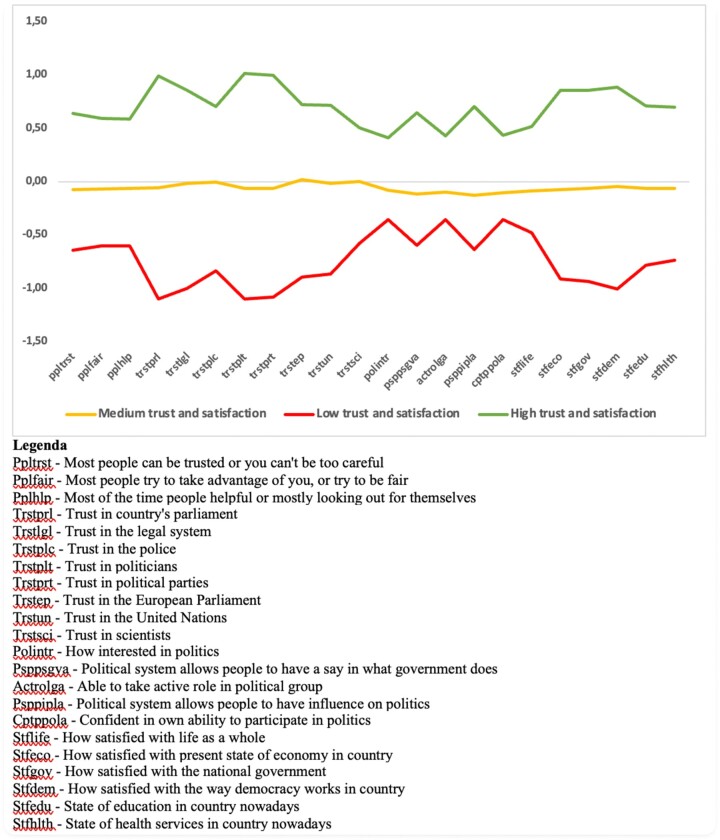
Graphical plot of the item response probabilities (*z*-scores) for the three latent classes (low, medium and high trust and satisfaction). Data source: ESS round 10, 2021.

The probability of belonging to the group of conspiracy believers is higher among men than women and in the middle age groups, when compared to the younger and older ones. Furthermore, this probability decreases as the educational level increases and the economic situation improves. Eventually, a greater inclination toward conspiracy beliefs is found among the unemployed and those who show low levels of trust and satisfaction. In addition, political factors are associated with conspiracy beliefs: right-wing orientations were associated with the endorsement of conspiracy beliefs (figure 2). The role of country of residence as contextual factors is confirmed, as the ICC show as 11% of the variance in the conspiracy index occurs at the country level. Empirical predictions of countries’ average probability of belonging to the latent class of conspiracy believers are reported in Supplementary figure S1.

### Step 3

Finally, the relationship between the conspiracy index and the main items of the COVID-19 module is analysed:

1. Is it more important to prioritize public health or economic activity when fighting a pandemic?
2. Is it more important for governments to monitor and track the public or to maintain public privacy when fighting a pandemic?
3. How satisfied you are with the way health services in [country] coped with the coronavirus pandemic and its consequences?
  Will you get vaccinated against coronavirus with the vaccine that was approved by the national regulatory authority in your country?

Questions a, b and c are asked using a Cantril scale ranging from 0 to 10. Table 2 shows the comparison in the various countries between the share of individuals placed in the left portion of the scale (scores from 0 to 4 for items a and b), in the right portion of the scale (scores from 6 to 10 for item c) and of individuals with a negative attitude toward vaccine (i.e. affirming they would/will not get vaccinated with a vaccine approved by the national regulatory authority in the country) out of the total within the two sub-populations represented by individuals classified as conspiracy believers with the previous LCA versus non-conspiracy believers.

**Table 2:** Percentage distribution of COVID items by conspiracy belief

	Public health is a priority (a)		Tracking is important (b)		Satisfied with health services (c)		Negative Vax_Attitude (d)	
Country	Conspiracy No (%)	Conspiracy Yes (%)	Conspiracy No (%)	Conspiracy Yes (%)	Conspiracy No (%)	Conspiracy Yes (%)	Conspiracy No (%)	Conspiracy Yes (%)
BG	51.0	49.9	23.9	22.2	45.0	23.7	50.4	69.7
CH	59.3	49.8	25.4	13.4	85.3	65.3	13.7	42.9
CZ	48.8	42.9	13.7	7.5	82.6	72.8	21.5	40.0
EE	48.5	52.0	22.7	13.8	70.6	44.9	17.3	34.5
FI	69.5	57.7	42.0	26.9	92.8	82.0	4.0	15.7
GR	70.6	36.4	32.5	13.5	50.0	21.6	9.6	28.6
HR	49.0	48.2	13.0	15.5	68.2	59.7	22.6	41.0
HU	54.4	60.8	17.6	18.1	57.1	57.0	17.6	38.0
IS	76.4	68.3	51.8	39.0	90.2	70.7	1.9	13.0
IT	54.2	51.4	47.1	45.1	60.0	52.4	3.5	6.9
LT	43.3	50.5	27.7	19.9	48.0	23.4	15.1	38.1
MK	47.9	52.2	24.6	36.3	38.4	29.6	22.0	26.7
NL	59.5	41.0	33.0	13.9	78.3	65.6	6.2	31.5
NO	68.2	61.0	36.5	40.4	93.7	80.5	4.3	21.4
PT	66.2	63.1	36.5	32.5	72.2	71.4	2.5	5.4
SI	63.0	52.7	33.8	32.1	57.3	39.5	31.1	46.4
SK	52.4	61.2	28.5	20.3	45.9	26.9	21.3	46.3
Total	57.3	51.4	35.5	27.7	66.5	51.0	9.7	27.6

Data source: ESS round 10, 2021.

Respondents who expressed conspiracy beliefs (WECB) have negative attitudes toward COVID-19 vaccines and are less satisfied with the way health services coped with the coronavirus pandemic as well as they would not support governmental restrictions. Considering the satisfaction with national health services, the gap between WECB and those who did not are larger in eastern European countries, where the conspiracy beliefs are more widespread. On the other hand, the differences of attitude toward governmental restrictions, which privilege tracking and public health priorities, are less marked between WECB and not WECB; for these aspects, the country factor prevails rather than adherence to conspiracy beliefs (table 2).

## Discussion

This study explores the role of personal attitudes and individual socio-demographic characteristics in determining COVID-19 conspiracy beliefs, as well as the relationship of COVID-19 conspiracy beliefs with both COVID-19 vaccine uptake and preferences about the policies adopted during the pandemic in 17 European countries.

Two LCAs were used to compute both a conspiracy index (based on 3 items from the ESS10) and a personal attitude index (based on 22 items from the same survey) for each responder. By using such indices, about 19% of the surveyed population was classified as conspiracist, while about 23% was found with lower levels of trust, interest in public life and overall satisfaction.

A multilevel weighted general linear regression model found an association between the conspiracy index and the attitude index, which was stronger for the class with the worst attitudes profile (i.e. with low levels of trust and satisfaction). The same model also found a positive relationship of conspiracy beliefs with male gender, middle age, lower educational level, worse perceived economic situation and being unemployed. Interestingly, in the multilevel model, the role of the country of residence as a contextual factor is confirmed (Supplementary figure S1).

Overall, respondents with conspiracy beliefs had lower levels of COVID-19 vaccine uptake (27.6% vs. 9.7% of those without such beliefs). This is consistent with results from previous studies, which found a relationship between conspiracy and vaccine hesitancy, both for COVID-19 and other infections.^7^^,^^26–32^ Similarly, a significant independent effect on vaccine acceptance was found in the previous editions of the ESS for trust in the scientific community, low participation in public life, right-wing political orientation and low education.^33^^,^^34^ Finally, a recent study on data from the 2019 Gallup survey found individuals’ trust in medicine and scientists and confidence in healthcare providers as predictors of vaccine acceptance, while also considering the concurrent effect of aggregate country-level wealth.^35^

Higher levels of conspiracy were related to lower levels of COVID-19 vaccine uptake in all surveyed countries. The difference between respondents WECB and other respondents was deeply higher in countries such as the Czech Republic (42.9% vs. 13.7%) and Slovakia (46.3% vs. 21.3%), while it was much less evident in other countries such as Portugal (5.4% vs. 2.5%) and Italy (6.9% vs. 3.5%). Overall, lower absolute levels of vaccine uptake were related with a larger difference between respondents WECB and the other respondents. This might suggest that in those countries where COVID-19 vaccination was only recommended or otherwise imposed less strongly, conspiracy beliefs might have had a stronger modulating influence on vaccination behaviour. Conversely, in those countries where COVID-19 vaccination was strongly nudged for the general population and even made compulsory for some categories (e.g. Italy), there might have been less space for the transformation of conspiracy beliefs into anti-vaccination behaviours.

The item concerning COVID-19 vaccination probably measured a mixture of intention to vaccinate and contextual-dependent vaccination behaviour, as the ESS10 survey was administered across heterogeneous timeframes and in many countries concluded long after the vaccination campaign had started. Consequently, while some respondents might actually have declared their intention to vaccinate or not, many of them have probably just declared their own vaccination status, which might have been influenced not just by their intentions but also by other factors (e.g. laws making the COVID-19 vaccination compulsory). This hypothesis seems to be supported by the similarity of respondents’ statement with respect to COVID-19 vaccination and official data on COVID-19 vaccination coverage from the ECDC (Supplementary table S3). For this reason, it was considered more appropriate for this study to mention the vaccine uptake, rather than the vaccine hesitancy (which is a more complex phenomenon, ranging from unsureness to delay to complete refusal).^36^

Having conspiracy beliefs was also associated with lower levels of agreement concerning: the importance to prioritize public health over economic activity when fighting a pandemic; the importance to prioritize monitoring and tracking activities over maintaining public privacy when fighting a pandemic; the satisfaction with the way health services coped with the pandemic and its consequences. These results replicate findings from a previous American study, demonstrating that participants who believed one or more conspiracies about COVID-19 expressed less support of policies to prevent the spread of COVID-19 (stay-at-home or shelter-in-place orders, cancelling or postponing mass gatherings, school closures, workplaces and businesses temporarily closing, travel bans and closures of non-essential businesses) than participants who disbelieved conspiracies.^32^

The difference between conspiracists and non-conspiracists was overall more evident for the item concerning the satisfaction with response of health services to the pandemic. This was also the only item of the three for which conspiracists had lower levels of agreement across all countries, with larger differences in Greece, Estonia, Lithuania and Bulgaria. The other two items showed a more heterogeneous relationship with the conspiracy index. As a matter of fact, higher agreement concerning the importance to prioritize public health over the economy was found among respondents WECB in Slovakia, Lithuania, Hungary, North Macedonia and Estonia. A similar result was found for the item concerning the importance to prioritize monitoring and tracking activities over privacy in North Macedonia, Norway, Croatia and Hungary. For these countries, individual conspiracy beliefs do not appear to be related to attitudes toward governmental restrictions, suggesting a stronger effect of other individual-level or contextual country-level variables. Further research to better outline this issue is needed.

To our knowledge, this is the first study to evaluate the prevalence and determinants of conspiracy beliefs simultaneously in 17 European countries, and to show an effect of conspiracy beliefs on the determination of COVID-19 vaccine behaviour and on the acceptance of the policies adopted during the pandemic.

The ESS10 data proved a valuable source for assessing not only the cross-national spread of conspiracy beliefs and attitudes, but also for estimating their possible public health impacts.

The choice of an LCA-based approach made it possible to synthesize a wide set of items to characterize respondents’ attitudes conspiracy beliefs in composite classes. This approach does not involve competition between indicators, unlike a purely regression-based approach would have done, avoiding the potential loss of significance that might have affected many relevant predictors of conspiracy with the latter approach.^37^^,^^38^ The subsequent multilevel regression analysis of conspiracy classes enabled a refinement of the picture regarding conspiracy class membership, which resulted influenced by other factors (i.e. socio-demographic variables, country of residence) other than attitude class membership. In particular, the advantage of this kind of regression, is to consider sources of variance both at the individual and at the ecological level, providing a more complete view of the situation and limiting the risk of confounding by spatial correlation and of ecological/sociological bias.

On the other hand, the appropriateness of a multilevel analysis is confirmed, as there is some evidence for a possible country contextual phenomenon shaping a common individual conspiracy level.^39^

This study has several limitations. First, all analyses were conducted on cross-sectional data, which does not allow making causal statements about ‘influence’, but only to determine associations between class membership and covariates or agreement to the four items of the COVID-19 module. Second, the survey was administered in different timeframes in different countries (Supplementary table S3), so the resulting data might not be fully comparable among countries. Third, even if the ecological influence of the country of residence was confirmed by the multilevel model, the sources of this kind of variability should be further clarified.

The present study highlights the importance of addressing the underlying attitudes and beliefs of individuals in promoting vaccine acceptance and ensuring public health measures are effective. Additionally, it suggests that there may be a country-level contextual influence in shaping a common individual conspiracy level, emphasizing the appropriateness of a multilevel analysis when investigating conspiracy beliefs. Understanding the contextual factors that influence conspiracy beliefs can assist in developing strategies to counter misinformation and improve public health communication. Furthermore, by understanding the relationship of vaccine uptake, acceptance of public health measures and conspiracy beliefs in different countries, public health officials can develop targeted interventions tailored to address the specific beliefs and attitudes of different populations and promote higher compliance in those populations.

## Supplementary Material

ckad089_Supplementary_Data

## Data Availability

The dataset was derived from sources in the public domain: ESS Data Portal, ESS10—integrated file (edition 2.2), available at https://ess-search.nsd.no/en/study/172ac431-2a06-41df-9dab-c1fd8f3877e7.

## Supplementary data

Key points

- The study examines personal attitudes and socio-demographic characteristics of individuals to determine COVID-19 conspiracy beliefs in 17 European countries.
- Latent class analyses were used to compute conspiracy and personal attitude indices for each responder, finding that 19% of respondents were classified as conspiracist.
- The study found a positive relationship between conspiracy beliefs and male gender, middle age, lower educational level, worse perceived economic situation and being unemployed.
- Higher levels of conspiracy were related to lower levels of COVID-19 vaccine uptake in all surveyed countries.
- Having conspiracy beliefs was associated with lower levels of agreement concerning the importance of prioritizing public health over economic activity, monitoring and tracking activities over maintaining public privacy and satisfaction with the way health services coped with the pandemic and its consequences.
