# Supplementary material for: Conspiracy beliefs, COVID-19 vaccine uptake and adherence to public health interventions during the pandemic in Europe
Source: Eur J Public Health. 2023 Jun 8;33(4):717–24. doi: 10.1093/eurpub/ckad089 (PMC10393495; doi:10.1093/eurpub/ckad089)

Supplementary materials

Table S1a. Conspiracy items by level of agreement, row percentages. Data source: ESS round 10, 2021

	Agree strongly	Agree	Neither agree nor disagree	Disagree	Disagree strongly	Refusal	Don't know	No answer	Total
Scientist deceive (a)	6.6	20.6	25.1	26.8	14.8	0.4	5.5	0.1	100
A small group (b)	8.9	23.2	23.8	22.2	14.7	0.5	6.7	0.1	100
Covid-19 is deliberate (c)	8.3	18.3	25.7	22.3	17.9	0.5	6.8	0.2	100

Table S1b. Frequency distribution of the variables identifying conspiracy attitudes by country (row percentages). Data source: ESS round 10, 2021

	Scientists deceive (a)			*A small secret group (b)*			*COVID is deliberate (c)*		
	agree	*neutral*	*disagree*	*agree*	*neutral*	*disagree*	*agree*	*neutral*	*disagree*
**BG**	47.9	32.0	20.1	56.3	27.8	16.0	57.9	27.8	14.4
**CH**	20.1	19.7	60.2	20.1	18.2	61.7	17.4	24.0	58.6
**CZ**	29.2	23.9	46.9	30.3	21.2	48.5	28.2	27.3	44.5
**EE**	29.2	30.4	40.4	24.9	26.6	48.5	27.3	30.3	42.4
**FI**	14.8	19.8	65.4	15.4	22.4	62.1	12.0	22.2	65.8
**GR**	21.6	24.9	53.5	35.9	22.9	41.2	22.0	22.5	55.5
**HR**	39.7	33.2	27.1	51.7	27.5	20.9	39.0	35.7	25.2
**HU**	28.3	34.1	37.7	34.7	32.4	32.9	28.1	33.7	38.2
**IS**	17.8	24.9	57.3	26.1	36.1	37.8	9.9	20.7	69.3
**IT**	20.4	29.9	49.8	26.8	28.6	44.5	25.3	33.7	41.1
**LT**	23.0	30.8	46.2	27.2	37.0	35.8	28.6	32.4	39.0
**MK**	49.5	30.6	19.9	59.1	24.9	16.0	53.6	29.0	17.5
**NL**	17.5	18.8	63.7	20.9	20.2	58.9	10.6	17.5	71.9
**NO**	15.5	28.0	56.5	12.3	24.5	63.1	10.3	23.3	66.4
**PT**	40.6	21.2	38.2	47.2	25.8	27.0	31.6	33.8	34.6
**SI**	38.4	28.8	32.7	50.2	23.4	26.4	35.1	30.0	34.9
**SK**	32.1	26.8	41.1	37.3	25.0	37.8	37.4	25.0	37.6

Table S2: Weighted selected descriptive statistics for ESS participating countries who filled the Covid module for Round 10 (row percentages).

Country	*N. of respondents*	*Gender*		*Age classes*					*Education*			*Occupational status*		
		M	*F*	*15-29*	*30-44*	*45-59*	*60-74*	*75+*	*Lower secondary or lower*	*Upper secondary*	*Tertiary*	*Employed*	*Unemployed*	*Inactive*
**BG**	2.718	47,9	52,1	17,5	23,3	24,0	23,6	11,5	25,0	51,6	23,4	50,7	7,8	41,5
**CH**	1.523	49,3	50,7	20,0	24,1	25,2	20,2	10,5	18,3	42,5	39,3	60,0	3,1	36,9
**CZ**	2.476	48,9	51,1	19,5	22,6	29,6	21,9	6,3	13,3	66,7	20,0	56,7	3,8	39,5
**EE**	1.542	46,4	53,6	17,7	25,7	24,1	22,0	10,4	14,9	47,9	37,2	61,9	3,3	34,8
**FI**	1.577	48,9	51,1	20,9	21,4	21,9	22,7	13,1	22,3	40,5	37,2	49,6	4,0	46,5
**GR**	2.799	48,6	51,4	15,5	22,5	28,9	23,1	10,1	34,0	40,6	25,5	52,1	7,7	40,2
**HR**	1.592	47,8	52,2	19,4	20,1	23,1	24,2	13,2	24,0	55,9	20,1	46,0	7,8	46,3
**HU**	1.849	47,3	52,7	19,9	22,9	26,2	20,7	10,3	23,4	53,3	23,3	56,6	3,3	40,1
**IS**	903	51,6	48,4	28,5	24,8	24,4	15,5	6,8	30,4	36,2	33,4	61,2	3,0	35,7
**IT**	2.640	48,3	51,7	18,0	18,7	26,7	22,3	14,3	47,5	36,7	15,8	48,1	8,2	43,7
**LT**	1.659	46,1	53,9	19,0	24,1	25,6	24,1	7,2	13,4	50,6	36,0	52,2	7,2	40,6
**MK**	1.429	50,1	49,9	19,9	35,9	26,5	12,7	5,0	23,9	55,7	20,4	51,9	19,5	28,6

**NL**	1.470	49,5	50,5		23,3	20,8	25,2	20,9	9,8		29,4	36,9	33,6		54,2	1,8	44,0
**NO**	1.411	51,7	48,3		23,9	23,8	25,2	19,3	7,8		21,8	40,3	37,9		57,8	2,0	40,3
**PT**	1.838	46,5	53,5		17,1	22,0	28,6	21,1	11,2		53,4	24,0	22,6		51,4	7,1	41,5
**SI**	1.252	50,0	50,0		17,5	23,1	28,2	21,2	9,9		17,6	52,2	30,3		54,3	4,0	41,7
**SK**	1.418	48,5	51,5		20,2	24,4	24,0	22,0	9,4		14,7	62,5	22,8		51,5	7,9	40,6

Table S3. Comparison of the percentages of respondents of the ESS who declared "they have been or will get vaccinated against COVID-19 with vaccine approved by the national regulatory authority in your country" and official data about vaccination status from Ourworldindata/ECDC at the time of the interview.

Country		Data from ESS survey					Official data		
		Yes, I will	*Yes, already*	*No*	*Data collection period*		*Fully vaccinated*	*Partially*	*Fully + Partially*
		%	*%*	*%*	***start***	***end***	*%*	*%*	*%*
**Bulgaria**	BG	15.9	18.5	65.7	28/06/2021	30/09/2021	19.7	0.0	19.7
**Switzerland**	CH	13.6	68.6	17.8	04/05/2021	02/05/2022	68.6	1.0	69.6
**Czechia**	CZ	13.2	59.4	27.3	07/07/2021	29/09/2021	57.0	1.1	58.1
**Estonia**	EE	10.0	69.0	21.0	07/06/2021	31/12/2021	61.0	2.0	63.0
**Finland**	FI	5.3	89.6	5.1	31/08/2021	31/01/2022	75.0	3.0	78.0
**Greece**	GR	6.3	80.6	13.1	09/11/2021	23/05/2022	73.0	2.8	75.8
**Croatia**	HR	18.5	50.4	31.1	05/05/2021	26/11/2021	48.0	6.0	54.0
**Hungary**	HU	8.1	68.7	23.1	10/06/2021	16/10/2021	57.0	2.2	59.2
**Iceland**	IS	2.8	94.4	2.8	28/07/2021	11/02/2022	77.0	5.7	82.7
**Italy**	IT	6.4	89.4	4.2	25/10/2021	26/04/2022	81.0	4.9	85.9
**Lituania**	LT	11.4	68.4	20.3	01/07/2021	15/12/2021	66.0	3.1	69.1
**Macedonia**	MK	10.3	64.5	25.2	23/10/2021	07/03/2022	40.0	1.0	41.0
**Netherland**	NL	8.0	83.2	8.8	01/10/2021	03/04/2022	68.0	4.7	72.7
**Norway**	NO	8.1	86.5	5.4	10/06/2021	04/05/2022	74.0	5.6	79.6
**Portugal**	PT	2.6	94.1	3.4	16/08/2021	06/03/2022	85.0	9.2	94.2
**Slovenia**	SI	17.3	46.1	36.6	18/09/2020	26/08/2021	43.0	4.4	47.4
**Slovakia**	SK	13.0	56.5	30.5	25/05/2021	21/10/2021	43.2	1.3	44.5

Table S4. Estimated class conditional probabilities for the three latent classes. Data source: ESS round 10, 2021

Conspiracy items	Class	Probabilities				
		Agree strongly	Agree	Neither agree nor disagree	Disagree	Disagree strongly
Small secret group of people responsible for making all major decisions in world politics	1	,013	,045	,071	,430	,441
	2	,346	,520	,067	,038	,028
	3	,019	,255	,502	,207	,017
Groups of scientists manipulate, fabricate, or suppress evidence in order to deceive the public	1	,005	,015	,041	,474	,466
	2	,285	,577	,097	,027	,014
	3	,001	,178	,533	,284	,004
COVID-19 is result of deliberate and concealed efforts of some government or organisation	1	,017	,037	,106	,358	,483
	2	,312	,455	,163	,044	,027
	3	,024	,178	,472	,261	,065

Figure S1. Empirical best linear unbiased country predictions of the probability of belonging to the latent class of conspiracy believers related to the overall mean

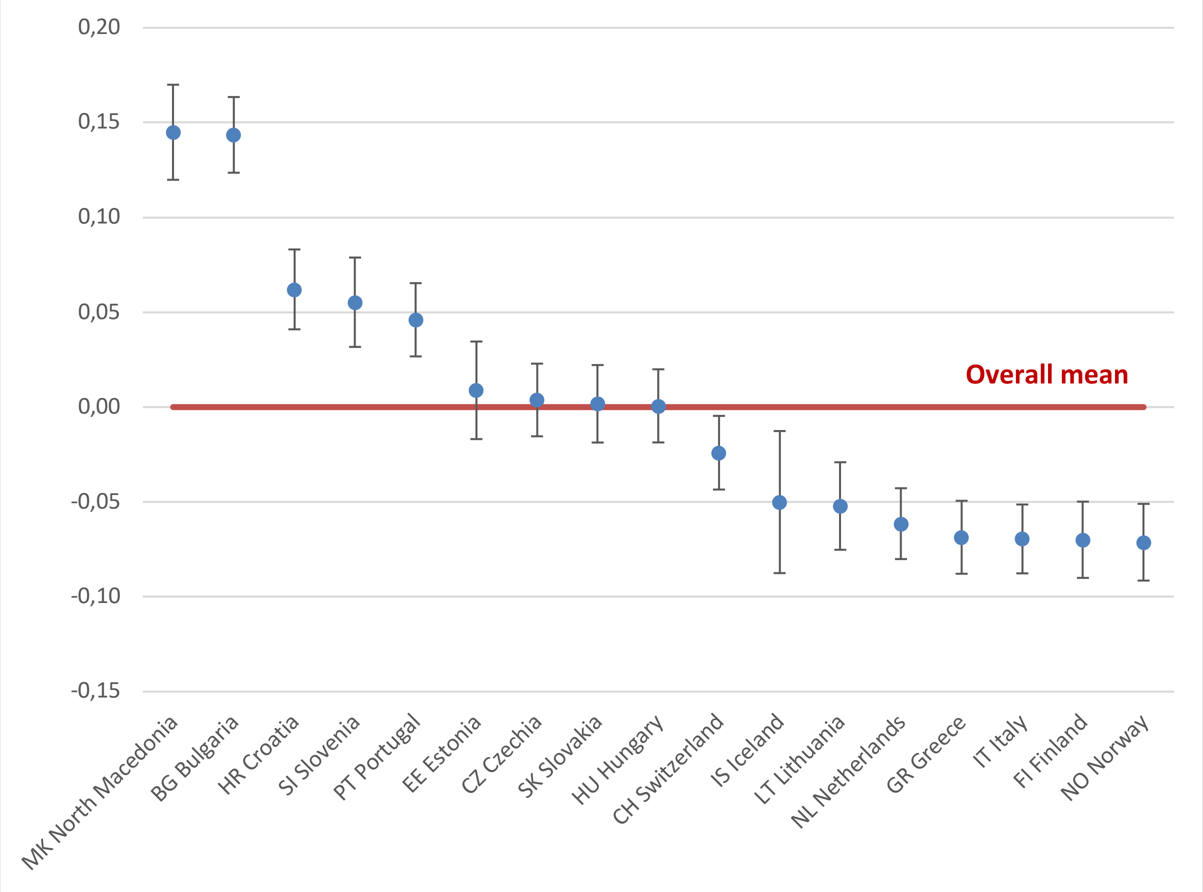

Supplement: ckad089_Supplementary_Data [file ckad089_supplementary_data.pdf]
